# Not a Stockholder

**Published:** 1893-12

**Authors:** 


NOT A STOCKHOLDER.—Our valued friend, Dr. W. C. Wile, of the *New England Medical Journal,* feels aggrieved at our surmise expressed, in a recent issue, that he was a stockholder in the company engaged in manufacturing "certain animal extracts" by Dr. Hammond's formula.

The warmth with which Dr. Wile flew to the defense of Dr. Hammond from an intimation that his—Dr. Hammond's—course with regard to the animal extracts had not been exactly in accordance with the JOURNAL'S idea of "legitimate medicine," and especially as he impugned the JOURNAL'S motives, warranted us, we thought, in the belief that he was an interested party. As the doctor has assured us, however, that we are mistaken, we take pleasure in so stating; we most assuredly would not knowingly do him a wrong.

